# Proteomic Signatures of Microbial Adaptation to the Highest Ultraviolet-Irradiation on Earth: Lessons From a Soil Actinobacterium

**DOI:** 10.3389/fmicb.2022.791714

**Published:** 2022-03-15

**Authors:** Federico Zannier, Luciano R. Portero, Thierry Douki, Wolfgang Gärtner, María E. Farías, Virginia H. Albarracín

**Affiliations:** ^1^Laboratorio de Microbiología Ultraestructural y Molecular, Centro Integral de Microscopía Electrónica, Facultad de Agronomía y Zootecnia, UNT y Centro Científico Tecnológico, CONICET NOASUR, San Miguel de Tucumán, Argentina; ^2^Laboratorio de Investigaciones Microbiológicas de Lagunas Andinas, Planta Piloto de Procesos Industriales y Microbiológicos, Centro Científico Tecnológico, CONICET NOASUR, San Miguel de Tucumán, Argentina; ^3^Université Grenoble Alpes, Commissariat a l’Energie Atomique et aux Energies Alternatives, Centre National de la Recherche Scientifique, Institut de Recherche Interdisciplinaire de Grenoble–Systèmes Moléculaires et nanoMatériaux p our l’Énergie et la Santé, Grenoble, France; ^4^Institute of Analytical Chemistry, University of Leipzig, Leipzig, Germany; ^5^Facultad de Ciencias Naturales e Instituto Miguel Lillo, Universidad Nacional de Tucumán, San Miguel de Tucumán, Argentina

**Keywords:** *Nesterenkonia*, soil bacteria, Puna, proteomics, extremophiles, UV

## Abstract

In the Central Andean region in South America, high-altitude ecosystems (3500–6000 masl) are distributed across Argentina, Chile, Bolivia, and Peru, in which poly-extremophilic microbes thrive under extreme environmental conditions. In particular, in the Puna region, total solar irradiation and UV incidence are the highest on Earth, thus, restraining the physiology of individual microorganisms and the composition of microbial communities. UV-resistance of microbial strains thriving in High-Altitude Andean Lakes was demonstrated and their mechanisms were partially characterized by genomic analysis, biochemical and physiological assays. Then, the existence of a network of physiological and molecular mechanisms triggered by ultraviolet light exposure was hypothesized and called “UV-resistome”. It includes some or all of the following subsystems: (i) UV sensing and effective response regulators, (ii) UV-avoidance and shielding strategies, (iii) damage tolerance and oxidative stress response, (iv) energy management and metabolic resetting, and (v) DNA damage repair. Genes involved in the described UV-resistome were recently described in the genome of *Nesterenkonia* sp. Act20, an actinobacterium which showed survival to high UV-B doses as well as efficient photorepairing capability. The aim of this work was to use a proteomic approach together with photoproduct measurements to help dissecting the molecular events involved in the adaptive response of a model High-Altitude Andean Lakes (HAAL) extremophilic actinobacterium, *Nesterenkonia* sp. Act20, under artificial UV-B radiation. Our results demonstrate that UV-B exposure induced over-abundance of a well-defined set of proteins while recovery treatments restored the proteomic profiles present before the UV-challenge. The proteins involved in this complex molecular network were categorized within the UV-resistome subsystems: damage tolerance and oxidative stress response, energy management and metabolic resetting, and DNA damage repair.

## Introduction

Free-living microorganisms endure physicochemical stochasticity of their environments, and due to their size and simplicity, they have relatively limited capabilities to adapt to or avoid the exposure of high levels of harmful environmental agents. Therefore, in unicellular organisms, genetic regulation plays a pivotal role in survival under stress conditions as it contributes to rescheduling gene expression and coordinating the synthesis of defense proteins, usually at the expense of expression of genes related to the cellular growth system. Changes in the concentration and activity of proteins encoded by these genes constitute the ‘adaptive response’ that, in turn, establishes a feedback circuit that modulates its intensity and duration, allowing the genetic system to restore the levels of expression before the onset of the stimulus or to adapt to the new environmental condition (Ramírez Santos et al., 2001; Aertsen and Michiels, 2004; Kaern et al., 2005). Thus, the adaptive response is a vital element for microorganisms that live in habitats with large physicochemical fluctuations allowing them to anticipate and acclimatize to harmful conditions (Thattai and Van Oudenaarden, 2004).

In the Central Andean region in South America, high-altitude ecosystems (3500–6000 masl) are distributed across Argentina, Chile, Bolivia, and Peru, in which poly-extremophilic microbes thrive under extreme environmental conditions (Albarracín et al., 2012, 2016; Orellana et al., 2018; Farías, 2020; Vignale et al., 2021). Previous studies demonstrated the high tolerance of these extremophiles to antibiotics, exposure to heavy metals and arsenic, dehydration, hypersalinity, and UV irradiation (Zenoff et al., 2006a,b; Albarracín et al., 2012, 2014; Dib et al., 2009; Flores et al., 2009; Belfiore et al., 2013; Kurth et al., 2015, 2017; Rascovan et al., 2016; Pérez et al., 2017; Rasuk et al., 2017; Ordoñez et al., 2018; Orellana et al., 2018; Portero et al., 2019; Zannier et al., 2019; Alonso-Reyes et al., 2020; Perez et al., 2020; Saona et al., 2021). Moreover, various underlying mechanisms and cellular processes were identified that indicate how these indigenous microbes cope with these multiple stress conditions, thus presenting an interesting side aspect of opening avenues of research for novel biotechnological applications.

In the Puna eco-region of the Andes between latitudes 8°S and 30°S, total solar irradiation and UV incidence are the highest on Earth (Liley and Mckenzie, 2006; Cabrol et al., 2009). UV measurements by climatologists and biologist exploring the Puna indicate that irradiance is 165% that of sea level with average UV-B values reaching 4 mW/m^2^ while short UV wavelengths incidence (260–270 nm) peaks at 14.6 mW/m^2^ on the ground (Liley and Mckenzie, 2006; Luccini et al., 2006; Cabrol et al., 2009, 2014). Also, both satellite-derived climatology as well as stations measurements register very high UV index and erythemal daily dose values with extreme monthly means above 18 and 10 kJ/m^2^ in December–January, respectively (Luccini et al., 2006). Moreover, UV levels in the Puna are considerably higher than those for equivalent regions in the Northern Hemisphere (Luccini et al., 2006), setting strong limits to the physiology of individual microorganisms and the composition of microbial communities in this eco-region (Cabrol et al., 2004, 2009, 2014; Zenoff et al., 2006a,b; Escudero et al., 2007; Flores et al., 2009; Ordoñez et al., 2009; Albarracín et al., 2011; Rascovan et al., 2016; Pérez et al., 2017; Toneatti et al., 2017). There is extensive research on these poly-extremophiles focusing on the influence of UV irradiation on the molecular profiles and adaptive strategies of model microorganisms isolated from shallow lakes and soil across different sites in the Argentinean and Chilean Central Andes (Albarracín et al., 2011, 2015, 2016). Genomics and ultrastructural and physiological assays identified a number of UV-resistance mechanisms of bacterial and archaeal strains (Zenoff et al., 2006a,b; Albarracín et al., 2012, 2014, 2016; Flores et al., 2009; Kurth et al., 2015; Rasuk et al., 2017; Toneatti et al., 2017; Portero et al., 2019; Alonso-Reyes et al., 2020, 2021).

Andean microbes’ high UV-resistance profile points to the existence of a network of physiological and molecular mechanisms triggered by ultraviolet light exposure. We called this the “UV-resistome” (Kurth et al., 2015; Portero et al., 2019; Alonso-Reyes et al., 2021) and it includes some or all of the following subsystems: (i) UV sensing and effective response regulators, (ii) UV-avoidance and shielding strategies, (iii) damage tolerance and oxidative stress response, (iv) energy management and metabolic resetting, and (v) DNA damage repair. Genes involved in the described UV-resistome were recently described in metagenomes (Kurth et al., 2017; Alonso-Reyes et al., 2020; Lamprecht-Grandío et al., 2020) as well as in many genomes from strains isolated from High-Altitude Andean Lakes (HAAL) including *Acinetobacter* sp. Ver3 (Kurth et al., 2015), *Salinivibrio* spp. (Gorriti et al., 2014), *Exiguobacterium* sp. S17 (Portero et al., 2019) and *Nesterenkonia* sp. Act20 (Alonso-Reyes et al., 2021). Among all these strains, we selected *Nesterenkonia* sp. Act20 as UV-resistant model strain to perform further assays. This selection is based on its superior UV-resistance profile (Rasuk et al., 2017; Portero et al., 2019) but also because of the high biotechnological potential of this actinobacterium. To our knowledge, there are not in-depth studies on the molecular basis of UV-resistance in this genus.

The aim of this work is to dissect the adaptive response of *Nesterenkonia* sp. Act20 upon UV-B using a proteomic approach together with photoproduct measurement. The molecular events during the UV challenge and after photorepairing were included within hypothesized UV-resistome subsystems, including DNA repair. Our results demonstrate that UV-B exposure induced over-abundance of an exclusive set of proteins while recovery treatments restored the proteomic profiles present before the UV-challenge.

## Materials and Methods

### Strains and Growth Conditions

*Nesterenkonia* sp. Act20 (strain Act20) belongs to the LIMLA extremophilic strain collection (Strain Number P156, PROIMI-CONICET). Axenic glycerol-freeze cultures were aerobically activated in a growth medium designed explicitly for this strain called “H medium” (i. e., Halophilic medium, 10 g NaCl, 3 g sodium citrate, pH 7–7.2) and cultured at 30°C with agitation (220 rpm) overnight. *Nesterenkonia halotolerans* DSM 15474 (strain DSM 15474), a close phylogenetic relative of strain Act20 but with a lower resistance profile to UV (Portero et al., 2019), was used as a control. This strain was grown under the same conditions as *Nesterenkonia* sp. Act20. Cultures of both strains were maintained in H agar (1.5%) for further inoculations.

### Preparation of Cultures for Photoproduct Measurements and Proteomic Analysis

*Nesterenkonia* sp. Act20 and *N. halotolerans* cultures were exposed to the following experimental conditions as described previously (Portero et al., 2019); Cell suspensions in NaCl 0.9% (20 ml, OD_600_ ∼0.6), exposed to artificial UV-B irradiation (5.4 W/m^2^ UV-B) for 20 min in quartz tubes (6.48 kJ/m^2^) using a Vilber Lourmat VL-4 lamp (maximum intensity at 312 nm) [Irradiance was quantified with a radiometer (09811-56, Cole Parmer Instrument Company, Vernon Hills, IL, United States)]. This dose reduces the viability of the strain Act20 to 50% compared to unexposed cultures (Portero et al., 2019). UV-treated subsamples were set aside for protein analysis without recovery treatment. This treatment was named “UV.” Subsequently, other UV-exposed subsamples were subjected to two parallel recovery treatments: named “photorecovery (“FR”)” and “dark recovery (“DR”) incubated under white light or in the dark for 120 min, respectively. On the other hand, a control treatment, named “total darkness” (“Dt”),” consisted of placing the cell suspensions in quartz tubes coated with aluminum foil and kept in the dark during the UV exposure and recovery treatments. An additional control for photoproduct measurements was provided by the non-exposed cell cultures at time 0 (T0).

All suspensions were then centrifuged, washed with NaCl (0.9%) and resuspended in 0.1 M Tris-HCl buffer, pH 7. The samples were stored at −70°C until subsequent processing. The above-described protocol was carried out in triplicate, obtaining three independent biological replicates for each treatment.

### Photoproduct Quantification

Ten milliliters of cell suspensions from the different treatments T0, Dt, UV, FR, and DR conditions were centrifuged at 3,000 × *g* for 10 min at 4°C. A cell suspension without exposure to any stimulus named T0 (initial time) was used as an additional control. Pellets were harvested and washed twice with distilled water. Total genomic DNA extraction was performed using a commercial genomic DNA kit (DNeasy Blood & Tissue Kit, Qiagen). Photoproducts were quantified using a pre-optimized procedure (Douki, 2013). After extraction, DNA was solubilized in an aqueous solution containing 0.1 mM desferrioxamine mesylate and then enzymatically hydrolyzed by incubation with nuclease P1, DNAase II, and phosphodiesterase II (2 h, 37°C, pH 6), followed by the second stage of digestion involving phosphodiesterase I and alkaline phosphatase (2 h, 37°C, pH 8). The digested DNA samples were injected into an Agilent 1100 Series HPLC system equipped with a reversed-phase ODB Uptisphere column (2 × 250 mm ID, particle size 5 μm, Interchim, Montluçon, France). The mobile phase (flow rate 0.2 ml/min) was an acetonitrile gradient (from 0 to 20%) in a 2 mM aqueous solution of triethylammonium acetate. The HPLC flow was split and funneled into an API 3000 electrospray triple quadrupole mass spectrometer operating in negative ionization mode. The pseudomolecular deprotonated ion of each photoproduct was collected and fragmented. Specific daughter ions of each photoproduct were quantified. Calibration curves were performed using proper reference compounds of varying concentrations. The results were expressed as the number of Photoproducts per 10^6^ DNA bases (bpm). The standards for HPLC-ESI-MS/MS were synthesized according to a previously published procedure (Douki and Cadet, 2001). In summary, dinucleoside monophosphates were prepared by the triester synthesis. Cyclobutane pyrimidine dimers (CPDs) were obtained by photosensitized triplet energy transfer using acetophenone and UV-A treatment. Pyrimidine (6-4) pyrimidone photoproducts (6-4PPs) were prepared by photolysis with UV-C irradiation, and a subsequent UV-A irradiation of these latter compounds produced the Dewar valence isomers (for a detailed photoproduct type description see Ravanat et al., 2001). All photoproducts were HPLC purified. The four *cis*-syn CPDs were measured [i.e., thymine-thymine (TTCPD), thymine-cytosine (TCCPD), cytosine-thymine (CTCPD), and cytosine-cytosine sites (CCCPD)], with cytosine under its deaminated form uracil. 6-4PPs at thymine-thymine (TT64) and at thymine-cytosine (TC64) sites, together with their Dewar valence isomers (DEWTT and DEWTC, respectively), were also quantified.

The above-described protocol was carried out as four individual set-ups, obtaining four independent biological replicates for each treatment and each photoproduct category. Significant differences in photoproduct absolute concentrations and mean efficiency on photoproduct repair were analyzed through an ANOVA model and TukeyHSD test. Photoproduct data are available in Supplementary file 3.

### Protein Extraction Protocol

Once the necessary three replicates of each treatment were obtained, we performed the protein extraction protocol described by Belfiore et al. (2013). Pellets were washed with Tris buffer (25 mM), pH 7, EDTA (2 mM) and then resuspended with the same buffer composition supplemented with 5 μl of a reducing solution (DTT 200 mM, Tris 100 mM, pH 7.8). Intracellular proteins were obtained by breaking the cells with a French press and incubating for 1 h at room temperature to achieve complete denaturation. The protein concentration was measured using the Bradford assay. For every 50 μg of protein, 20 μl of reducing solution and 20 μl of alkylating solution (iodoacetamide, 200 mM, Tris, 100 mM, pH 7.8) were added to each sample. The mixture was allowed to incubate and centrifuged at 16,100 × *g* for 30 min (4°C). Then, the proteins were precipitated with 10% TCA and incubated overnight (−20°C). Samples were centrifuged (16,100 × *g*, 30 min, 4°C), and the protein pellets were washed twice by rinsing with 500 ml pre-chilled (−20°C) acetone. Once dried, the protein pellets were dissolved in ammonium bicarbonate (50 mM) and digested with trypsin at 37°C for an incubation period of 14–16 h. Finally, after digestion with trypsin, the concentration of peptides was determined, and the samples were stored at −80°C until further analysis through mass spectrometry (MS).

### Protein Identification and Mass Spectrometry

Global proteomics (“Shotgun proteomics”) was performed using the “bottom-up” method following Bonilla et al., 2020. For this purpose, the protein samples were digested with trypsin and then cleaned with Zip-Tip C18 to extract salts. Then, liquid chromatography was performed with nanoUHPLC Easy nLC 1000 (Thermo Scientific brand, model EASY-nLC 1000, Easy-Spray ColumnPepMap RSLC, P/N ES801) (Thermo Scientific) coupled to a mass spectrometer with Orbitrap technology, which allows a separation of the peptides obtained by tryptic digestion of the sample and subsequent identification. Sample ionization was carried out by nanoelectrospray (Thermo Scientific brand, model EASY-SPRAY. Spray voltage: 1.8 kV). The instrument was equipped with an HCD (High Collision Dissociation) cell and an Orbitrap analyzer yielding the identification of peptides simultaneously to their separation by chromatography. The parameters used during the mass spectrometry analysis were based in full MS (MS1) scan followed by MS/MS (MS2) scans (Data Dependent Acquisition or DDA) to identify + 1 or multiply charged precursor ions in the mass spectrometry data file. MS1 and MS2 peak ranges were 300–1,800 Da (resolution 70000) and 65–2,000 Da (resolution 17500), respectively. The analysis of the raw files delivered by the mass spectrometer was performed using the ProteomeDiscoverer version 2.1 search engine through SEQUEST HT, conducting peptide-to-spectrum mapping (PSMs) against the sequenced genome of *Nesterenkonia* sp. Act20. The following parameters were set for the search: carbamidomethyl (C) on cysteine was set as fixed; variable modifications included asparagine (N) and glutamine (Q) deamidation and methionine (M) oxidation. Only one missed cleavage was allowed; monoisotopic masses were counted; the precursor peptide mass tolerance was set at 10 ppm; fragment mass tolerance was 0.05 Da. The MS2 spectra were searched with Proteome Discoverer v2.1 using a 95% confidence interval (CI%) threshold (*P* < 0.05). The described protocol was carried out at CEQUIBIEM (by its Spanish acronym, “Centro de Estudios Químicos y Biológicos por Espectrometría de Masa”), University of Buenos Aires-Argentina. ProteomeDiscoverer results are available in Supplementary file 1, sheet 1. Proteomic mass spectrometry raw files have been deposited at the Mass Spectrometry Interactive Virtual Environment repository^1^ with the dataset identifier MSV000088619.

### Statistical Data Analysis

Data analysis was performed using the software Perseus v.1.6.2.3, Microsoft Excel, R v.3.6.1, and Cytoscape v.3.7.1 (Shannon et al., 2003; R Core Team, 2016; Tyanova et al., 2016). Proteins with only one valid value in each treatment, in only one treatment, or with missing values in all three replicates in each of the four treatments were filtered out, selecting proteins with at least two valid values in at least one treatment. Next, log2 transformation was performed and missing values (NaN, “Not a number”) were imputed by the minimum detected values of the normal distribution of the whole dataset (Lazar et al., 2016) (Width = 0.5, Down shift = 1.8). Next, the abundance averages for each protein were compared between treatments through the *t*-test. Proteins were considered significantly regulated when (Witten and Tibshirani, 2007): (A) The *t*-test showed a value of significance *p* < 0.05, (B) the value of the difference between the log-scale averages of abundance (log2) was above or below the fold change (FC) 1 and −1 (−1 < FC_*difference*_ < 1), respectively (see note 1 in the Supplementary file 2). Treatments were compared against each other providing different information about the system under study (see section “Statistical Analysis of Proteomic Profiles Among Treatments”).

### Annotation and Bioinformatics Analysis

The sequenced genome of *Nesterenkonia* sp. Act20 has been deposited at DDBJ/ENA/GenBank under the accession JADPQH000000000 (Alonso-Reyes et al., 2021). The functional analysis of the identified proteins was performed using tools from the “KEGG” (Kyoto Encyclopedia of Genes and Genomes) and String databases (Szklarczyk et al., 2017) and exhaustive text mining.

## Results

### DNA Repair Ability of *Nesterenkonia* sp. Strains

Ultraviolet-resistance was herein indirectly explored by assessing the level of DNA damage after UV and its repair in Act20 and DSM 15474 strains. DNA photoproducts were measured by HPLC-ESI-MS/MS in UV-exposed cells and in cultures subjected to recovery treatments in light (FR) and dark (DR), respectively. DNA photoproducts were also quantified in unexposed samples at the beginning (T0) of the experiment.

**FIGURE 1:**
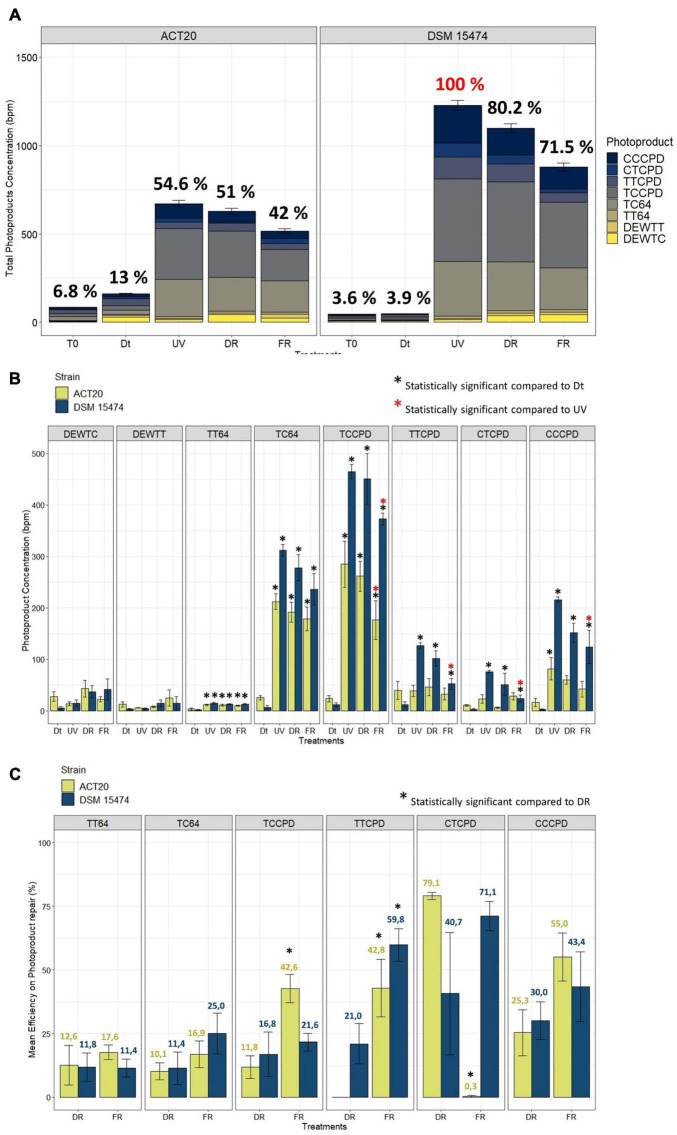
**(A)** Stacked barplot showing the mean total concentration [as bases per million (bpm)] of photoproducts generated by each treatment and the contribution of each photoproduct category. Error bars means the standard deviation of the cumulative sum of photoproducts within each treatment. The 100% of photoproduct (in red) able to be produced under our experimental conditions was attributed to the condition with the highest photoproduct concentration found in the UV-treated cultures of DSM 15474. Relations to the 100% are shown as percentages in black. **(B)** Quantification of *cis*-syn cyclobutane pyrimidine dimers (TTCPD, TCCPD, CTCPD, and CCCPD), pyrimidine (6-4) pyrimidone photoproducts (TT64 and TC64), and Dewar valence isomers (DEWTT and DEWTC) plotted as the mean of their absolute concentration (bpm) achieved in each treatment (Dt, UV, DR, and FR). **(C)** Comparisons of the mean efficiency of DNA photoproduct repair between DR and FR treatments. *Nesterenkonia* sp. Act20 (yellow bars), *Nesterenkonia halotolerans* DSM 15474 (blue bars). Asterisks indicate statistically significant differences (see legends in the picture).

The sum of photoproducts of all categories accumulated within each treatment showed the damage/repair balance on DNA in both strains (Figure 1A). The maximum accumulation of photoproducts was found in cultures of the strain DSM 15474 exposed to the UV treatment (1228 bpm). In accordance with its higher UV-resistance profile (Portero et al., 2019; Alonso-Reyes et al., 2021), the strain Act20 accumulated 45% less DNA photoproducts (671 bpm) than the strain DSM 15474 (1228 bpm) under UV-exposure. Similar patterns were also detected under DR and FR treatments, in which the strain Act20 accumulated ca. 627 and ca. 515 photoproducts per million bases, that is ca. 41% fewer photoproduct accumulation compared to the strain DSM 15474 exposed to DR (ca. 1098 bpm) and FR (ca. 879 bpm), respectively (Figure 1A).

Ultraviolet irradiation increased the abundance of all types of photoproduct categories except Dewar isomers (Figure 1B). In both strains, and in all conditions tested, the main types of photoproduct produced were TCCPD and TC64 (Figure 1A). However, there were differences observed between strains. Compared to unexposed cells, UV treatment significantly increased the accumulation of TT64, TC64, TCCPD, TTCPD, CTCPD, and CCCPD photoproduct in the strain DSM 15474, while induced significant accumulation of TT64, TC64, TCCPD, and CCCPD in the strain Act20. Interestingly, FR significantly decreased the absolute abundances of TCCPD, TTCPD, CTCPD, and CCPD measured in the strain DSM 15474 while significantly reduced TCCPD in the strain Act20.

The mean efficiency to repair TT64, TC64, TCCPD, TTCPD, CTCPD, and CCCPD photoproducts under DR and FR were estimated for both strains (Figure 1C). Repair of photoproducts increased always under FR, although those differences were not always statistically significant. Thus, the strain Act20 repaired TCCPD and TTCPD significantly better under light (42.6% and 42.8%) than under dark conditions (11.8% and 0%). Likewise, the strain DMS 15474 repaired TTCPD more efficiently under light (59.8%) than in the dark (21%). Also, both strains improved their repair efficiency for TC64 and CCCPD in the light rather than in the dark, although these differences are not statistically significant. Surprisingly, in the strain Act20, CTCPD were better repaired under dark recovery while under light this activity is scarce or even not detected (Figure 1C). Since photolyases are the only light-driven enzymes capable of repairing DNA damaged nucleotides, the CPD and 6-4 photolyases detected in the genomes of both Act20 and DSM15474 strains (Alonso-Reyes et al., 2021) may be responsible for the observed decrease in photoproduct concentration under FR. Therefore, photolyases may contribute to protecting the integrity of the genome and as such they constitute active elements of the UV-resistome in both strains.

### Panoramic Approach and Functional Orthology of Act20 Proteome

In order to explore the molecular basis of the superior UV-resistance profile of the strain Act20, we performed a proteomic study under four experimental conditions, Dt, UV, DR, and FR. Taking together the number of proteins with at least one valid value in at least one biological replicate of each treatment, a set of 1597 different proteins were detected, herein called the “experimental proteome (Ex.P),” that covers 59% of the predicted ORFs contained in the genome of the strain Act20 (Predicted proteome, Pred.P = 2689 ORFs). In this section, we performed a functional panoramic analysis of the proteomic profiles detected under each treatment based on detection limits of the mass spectrometry device. Thus, those proteins with missing values in the three samples of a treatment were considered as not present or deregulated under that condition. Contrary, the detection of proteins with at least one valid value in at least one sample of a treatment were considered as present in that treatment. Thus, by applied these criteria, there were 1476, 1521, 1546, and 1565 proteins listed in Dt, UV, DR, and FR proteomic datasets, respectively (Table 1 and Supplementary file 1, sheet 2).

**TABLE 1:** Summary of the number of proteins identified by mass spectrometry and the KEGG database for each conditions.

	Pred.P	Ex.P	Dt	UV	DR	FR
n° of proteins identified by MS	2689	1597	1476	1521	1546	1565
n° of proteins assigned to a KO	1381	1001	940	966	979	985
n° of different KO	1168	910	857	883	890	896
n° of pathways identified by KEGG server	155	150	149	150	150	150
n° of modules identified by KEGG server	38	37	33	37	37	36

Metabolic pathway and functional modules are sets of proteins that functionally interacts, either by catalyzing steps in a series of ordered biochemical reactions to yield metabolites or by integrating protein complexes linked to specific tasks, in which each protein is necessary and usually must be present to ensure the activity of a cellular process. To assess the complete metabolic pathways and functional modules in which the list of proteins present in Pred.P, Ex.P, Dt, UV, DR, and FR datasets are involved, their amino acids sequences were BLAST against KEGG database. KEGG assigns a code (KO) to each recognized protein according to its function. Several proteins can share the same KO code, but each protein is assigned to only a single KO that reflects its functionality. The number of different proteins and different functionalities carry out by them, as well as the number of metabolic pathways and functional modules identified by KEGG in each dataset are summarized in Table 1. The lowest number of functionalities and complete metabolic pathways as well as functional modules were observed in Dt dataset (Table 1).

**FIGURE 2:**
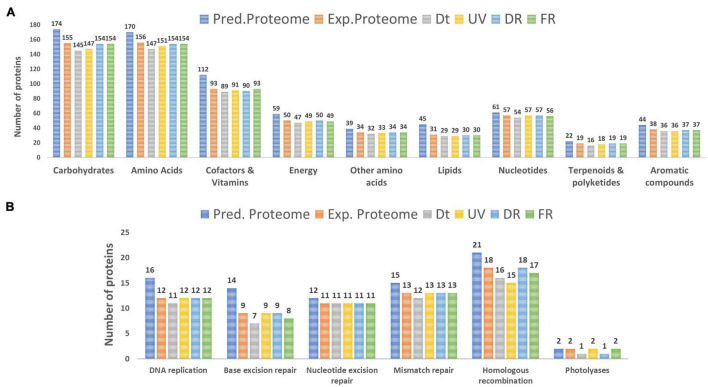
**(A)** Number of proteins involved in the metabolism of the main categories of biomolecules identified in the predicted proteome (Pred.P), the experimental proteome (Exp.P), Dt, UV, DR, and FR datasets. **(B)** Number of proteins involved in the main categories of DNA repair mechanisms.

To further assess the influence of each treatment on the metabolism of *Nesterenkonia* sp. Act20, the number of proteins involved in the main nine categories of biomolecules was counted. There were no major variations between treatment datasets and, also, there were high percentages of proteins shared among them for each particular category (>90%), indicating a low degree of protein replacement induced by each experimental condition (Figure 2A and Supplementary Table 1). Thus, the influence of treatments on the metabolism of the strain Act20 is not dependent on the expression of large groups of proteins, but rather indicate that the high shared number of proteins constitute a protein repertoire essential to survival while the different treatments can only change a small proportion of this essential protein base. Moreover, the reconstruction of functional modules using the KEGG server showed that the non-oxidative phase of the pentose phosphate cycle and the biosynthesis of ornithine from glutamate could be interrupted in the Dt treatment due to the deregulation (non-detection) of specific proteins [i.e., ribulose-phosphate 3-epimerase (EC:5.1.3.1) (rpe) (see Supplementary Figure 1 in Supplementary file 2), amino-acid *N*-acetyltransferase [EC:2.3.1.1] (ArgA), acetylornithine/*N*-succinyldiaminopimelate aminotransferase [EC:2.6.1.11 2.6.1.17] (ArgD)], as suggested by the mass spectrometer detection threshold (Supplementary file 1,sheet 2). In contrast, the set was proteins involved in these functional modules were detected in UV, DR, and FR protein datasets, respectively, suggesting that such metabolic processes could be influenced by UV exposure. Further, no proteins from the flagellum biosynthesis machinery were detected in Ex.P despite being encoded in the genome (Pred.P) (Supplementary file 1), which is consistent with the observation that no flagella were detected by electron microscopy in the strain Act20 with or without UV exposition (Alonso-Reyes et al., 2021). Also, specific pathways of carbohydrate metabolism involving the beta-*N*-acetylhexosaminidase [EC:3.2.1.52, (K01207)] (nagZ) protein could be affected by UV exposure as this protein was not detected in Dt but was detected in UV, DR, and FR (Supplementary file 1).

The DNA repair/metabolism subsystem of the hypothesized UV-resistome, including DNA replication, base excision repair, nucleotide excision repair, mismatch repair, homologous recombination and photolyases, was herein analyzed. In general, repairing treatments displayed more proteins for each of these repair functions than those in the control or the UV-treatment (Figure 2B). In fact, there was a probable variation in the number of proteins involved in homologous recombination among treatments. Remarkable, variations in the photolyase category agrees with the biological functionalities of these enzymes, as during the UV and FR treatments two types of photolyases, CPD as well as 6-4 photolyases, were expressed, while in the control (Dt) or under DR only CPD photolyases were present as constitutive photorepairing proteins (Figure 2B and see Supplementary file 1).

### Statistical Analysis of Proteomic Profiles

**FIGURE 3:**
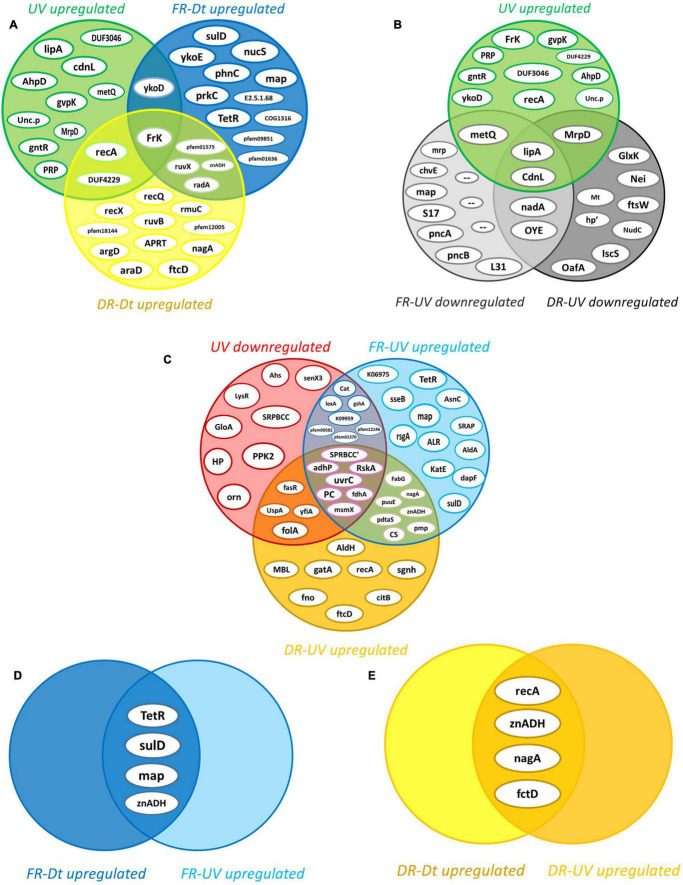
Intersection analysis of proteins upregulated by the effect of each treatment. **(A)** Intersection of the 14 UV-upregulated proteins (from UV-Dt) versus (vs.) the set of FR- and DR-upregulated proteins (from FR-Dt and DR-Dt comparisons) highlights those UV-induced proteins that remains significantly upregulated throughout the recovery treatments. **(B)** Intersection of UV-upregulated proteins from UV-Dt comparison vs. UV-upregulated proteins from FR-UV and DR-UV comparison highlights the set of UV-induced proteins whose abundance levels are most strongly increased by UV exposure. **(C)** Intersection of UV-downregulated proteins (from UV-Dt comparison) vs. FR- and DR-upregulated proteins (from FR-UV and DR-UV comparison) highlights the set of proteins whose abundance decreased under UV but then restored their levels dependently or independently of FR treatment. **(D)** Intersection of FR-upregulated proteins from FR-Dt vs. FR-UV highlights the set of proteins whose abundances are strongly induced by FR as they reach higher levels of abundance in FR than in Dt and UV. **(E)** Intersection of DR-upregulated proteins from DR-Dt vs. DR-UV highlights the set of proteins whose abundances are strongly induced by DR as they reach higher levels of abundance in DR than in Dt and UV.

By filtering and imputing the entire dataset there were 1522 out of the 1597 proteins with sufficient valid values to be analyzed by statistical methods (Lazar et al., 2016) (see section “Statistical Data Analysis”). The average abundance values of each protein were compared between treatments by the *t*-test, and the statistical significance of the FC_*difference*_ was estimated. The effect of artificial UV-B light exposure over the proteomic profile of the strain Act20 was determined mainly by comparing the UV treatment dataset against that of the Dt treatment (UV-Dt), although FR-UV and DR-UV comparisons were useful to interpret and to complete the UV-response model. Likewise, the effect of the photorecovery treatment was determined by comparing the abundance of proteins listed in FR dataset against Dt and UV (FR-Dt and FR-UV), and, in turn, by comparing each set of FR-upregulated proteins against UV-upregulated and UV-downregulated proteins (from UV-Dt). Also, DR-upregulated proteins taken from DR-Dt and DR-UV comparisons were added to the analysis in order to discriminate between FR-dependent and FR-independent effects on the proteomic profiles. Each particular comparison provides different information about the system under study (see Figure 3).

By applying the statistical criteria A and B (see section “Statistical Data Analysis”), there was a specific set of fourteen upregulated proteins induced by the UV challenge (Figure 3A). Of these, Fructokinase (EC 2.7.1.4) (FrK) remained upregulated after both recovery treatments, while Recombinase A (RecA), a DUF4229-domain-containing protein (DUF4229), and the HMP/thiamine import ATP-binding protein (YkoD) maintained significantly high levels of abundance during DR and FR, respectively (Figure 3A). Likewise, Lipoyl synthase [EC:2.8.1.8] (lipA) and the CarD N-terminal-like transcriptional regulator (CdnL) reached the highest abundance values during UV irradiation (Figure 3B), suggesting that they are relevant components of the UV-resistome of Act20 strain.

In contrast, 26 proteins were downregulated by UV exposure according to criteria A and B (Figure 3C). Among them, the abundance of the anti-sigma K factor (RskA), the Excinuclease C from UvrABC system (UvrC), 1,3-propanediol dehydrogenase (EC 1.1.1.202) (AdhP), Pyruvate carboxyl transferase (EC 6.4.1.1) (PC), glutathione-independent formaldehyde dehydrogenase [EC:1.2.1.46] (fdhA), the multiple sugar transport system ATP-binding protein (msmX), and an uncharacterized SRPBCC-domain-containing protein (SRPBCC’) were decreased during UV but then significantly increased under both recovery treatments after UV exposure (Figure 3C), reaching mean abundance values non-significantly different to those achieved in Dt conditions (see Supplementary file 1). Due to their levels were restored under FR as well as DR conditions after UV, this suggest that their regulation is independent on FR.

Contrary, the SOS-response repressor and protease LexA (EC 3.4.21.88) (lexA), the Mn-containing catalase (Cat), a glutamate—cysteine ligase/carboxylate-amine ligase (gshA), an hypothetical protein which KO code is K09959 as well as three uncharacterized proteins related to the universal stress (pfam00582), the nucleoside-diphosphate-sugar epimerases (pfam01370), and the serine aminopeptidases (pfam12146) protein families, recovered their abundance dependent on FR as this does not occur under the DR treatment (Figure 3C and Supplementary file 1).

On the other hand, among the proteins that increased their abundance under FR after UV exposure, there are a well-defined set of twelve proteins whose levels seems to be specifically induced by the FR treatment (Figure 3C). Of these, the bifunctional enzyme 2-amino-4-hydroxy-6-hydroxymethyldihydropteridine pyrophosphokinase (EC 2.7.6.3) (SulD) involved in folate synthesis, a transcriptional regulator from TetR family (TetR), the methionine amino peptidase (map), reached abundance values that significantly exceed those achieved under Dt and UV (Figure 3D), suggesting that they might be central elements of cell recovery under FR. The same is true for the zinc-dependent aldehyde dehydrogenase (znADH) which reached higher abundance in FR than under Dt and UV, although is also induced under DR (Figures 3C,D).

Altogether, these results indicated that UV-exposure triggered the upregulation of fourteen main proteins. Also, suggest that once the UV challenge ceased, some UV-downregulated proteins restore their abundance levels independent as well as dependently on the photorecovery treatment (FR), reaching non-significantly different values than those achieved under Dt conditions.

### Molecular Response Model Against Artificial Ultraviolet-B Radiation

**FIGURE 4:**
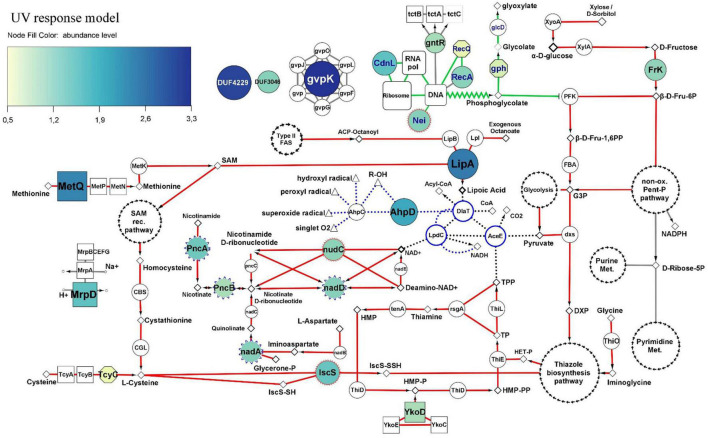
Molecular UV-response model of Act20 strain. Nodes of the DNA repair and metabolic resetting functional module are highlighted with blue labels and green interactions (borders). Interactions of the functional module related to cofactor biosynthesis for the PDH complex are highlighted in red. PDH complex proteins and their interactions are highlighted with blue edges nodes and black punted borders. The antioxidant redox interactions between free radicals, and AhpC, AhpD, DlaT, and LpdC proteins are represented as blue punted borders. Additional interactions with less relationship to the mentioned functional modules are painted in gray. The color and size of nodes representing significant UV-upregulated proteins indicates their abundance levels (see color coding at the top left corner). Color-less nodes indicate functionally related proteins encoded in the genome of strain Act20, but showing no significant UV-related changes in abundance. The size of these color-less nodes are independent of their abundance levels. Rectangular nodes represent membrane transporters. Nodes with blue scalloped edges (e.g., nadD, in center) and nodes with red edges (e.g., IscS and nudC, center bottom), represent proteins taken from the FR-UV and DR-UV comparisons (Figure 3B), respectively. White nodes with separate black arrow edges represent metabolic pathways. Diamonds, triangles, and small circles nodes, represent substrates, free radicals and ions, respectively. Functional interactions among nodes are represented by borders: metabolic pathways/proteins–substrates interactions are represented by solid lines; metabolic pathways/proteins–products interactions are represented by arrows; truncated lines represent inhibitions; zig-zag lines represent DNA repair derivative products. This schema does not consider reversible reactions. All proteins are annotated in Supplementary file 1, sheet 5.

In order to build a model of the molecular events involved in the response against UV, each of the main fourteen UV-upregulated proteins (Figure 3A) was functionally linked to a particular cellular process, when possible. Both the functions and biological implications of each protein were analyzed through extensive literature mining of homologous proteins using cured databases. In addition, to gain more evidence of the molecular events in which the proteins are involved, functionally related proteins (from UV-Dt comparison) with significant FC_*difference*_ but below the imposed threshold were analyzed and taken into account for the model. Also, significantly UV upregulated proteins from FR-UV and DR-UV comparisons were added (Figure 3B). Finally, in order to interpret and complete the model, functionally related proteins encoded in the genome of strain Act20, but showing no significant UV-related changes in abundance were also added. Through this analysis it was interpreted that the fourteen co-expressed proteins could be functionally linked to the hypothesized UV-resistome subsystems (Figure 4).

A first functional module was associated to DNA repair and metabolic resetting. We observed that RecA was significantly upregulated by the UV effect (Figure 3A), but also, its associated protein, the ATP-dependent DNA helicase RecQ [EC:3.6.4.12] (RecQ), presented significant increases of their abundance in UV than in Dt (Figure 4), although the FC_*difference*_ was lower than the threshold (criterion B) (Supplementary file 1 and Supplementary Figure 4). However, the comparison of the proteomic profiles achieved under the recovery treatments against the control condition also showed significant increases of other recombinant proteins (Figure 3A and Supplementary Figure 4). Among them, RecA, RecQ, and recombinant regulatory proteins that modulates RecA activity through direct physical interaction such as radA, recX, and rmuC, as well as proteins that promotes strand exchange reactions in homologous recombination such as the holliday junction DNA helicase RuvB [EC:3.6.4.12] (RuvB) and other putative holliday junction resolvase [EC:3.1.21.10] (RuvX) (Figure 3A), reached significantly higher abundance in DR than in Dt according to criteria A and B, as well as significant higher levels in FR but with slightly lower FC_*difference*_ compared to DR, respectively (Supplementary Figure 4). Thus, this notable induction of recombinant proteins abundance observed in UV-treated cultures suggest high activity levels of RecA-dependent processes and homologous recombination, an error-free repair system of single strand gaps and double strand breaks on DNA (Smith and Wang, 1989; Kowalczykowski et al., 1994; Nickoloff and Hoekstra, 2001; Khan and Kuzminov, 2012; Kreuzer, 2013).

Also, the formamidopyrimidine-DNA glycosylase with endonuclease VIII activity [EC:4.2.99.18] (Nei), involved in excising pyrimidines damaged by mutagenic agents (Palmer et al., 1997), showed higher levels during UV exposure than after the dark recovery treatment (Figure 3B), suggesting that it may be involved in the UV response (Figure 4). Furthermore, another protein that gives indirect evidence about the repair of damaged DNA caused by UV-induced oxidative stress is the upregulation of phosphoglycolate phosphatase [EC:3.1.3.18] (gph), involved in the dissimilation of intracellular 2-phosphoglycolate formed during the repair of the 3′-phosphoglycolate ends in the DNA (Pellicer et al., 2003). Its abundance value was significantly increased in UV compared to Dt but with FC_*difference*_ slightly lower than the threshold (Figure 4 and Supplementary file 1). This protein works together with glycolate oxidase [EC:1.1.3.15] (glcD) that reduce the glycolate yielded by gph to produce non-toxic glyoxylate and H_2_O_2_ which then can be removed by antioxidant enzymes (Pellicer et al., 2003). In addition, 2-phosphoglycolate is a potent inhibitor of 6-phosphofructokinase (EC 2.7.1.11) (PFK) impairing fructose-1,6-bisphosphate production (Seal and Rose, 1987; Figure 4).

Also, UV-induced metabolic resetting could be inferred due to the significant increase on CdnL abundance levels during UV exposure, as this protein has been linked to the global control of gene expression through a RNAP–protein interactions mechanism dependent on σA factor; its activity is vital and has been well characterized in Mycobacteriales (Stallings et al., 2009; Weiss et al., 2012; Srivastava et al., 2013; Garner et al., 2014, 2017; Kaur et al., 2014, 2018; Zhu et al., 2019) and Myxococcales (García-Moreno et al., 2010; Gallego-García et al., 2014; Bernal-Bernal et al., 2015) under different stress and virulence conditions. It is known that CdnL activates the transcription of rRNA and components of the transcription machinery (Garner et al., 2014, 2017). Although no measurements of ribosomal RNA levels were made in this study, the abundance of a set of ribosomal proteins, whose abundance are proportionally to rRNA expression levels (Garner et al., 2014, 2017), were significantly higher in the UV dataset compared to FR suggesting that ribosomal assembly and metabolic rates (Nomura, 1999; Casati, 2004) may be altered under UV exposure (Supplementary Figure 2 in Supplementary file 2).

A second functional module induced by UV was linked to the antioxidant activity of the pyruvate dehydrogenase complex (PDH) and to proteins that we interpret, could act systematically to provide the necessary substrates for the synthesis of the cofactors required by PDH. In this sense, PDH are enormous protein complexes containing many copies of three proteins named E1 (AceE), E2 (DlaT), and E3 (LpdC) and three cofactors, lipoic acid (lipoate), thiamine pyrophosphate (TPP) and NAD+ (Rodionov et al., 2002, 2004, 2008; Cicchillo et al., 2004; Spalding and Prigge, 2010; McCarthy and Booker, 2017). In addition, PDH can be associated with adaptor proteins and act as a powerful antioxidant complex that can efficiently eradicate free radicals generated by UV radiation (Bryk et al., 2002; Jaeger et al., 2004; Spalding and Prigge, 2010; Lu and Holmgren, 2014; Figure 4).

Lipoyl synthase (lipA) was highly upregulated by UV. This key enzyme of the lipoate synthesis pathway catalyzes sulfur insertion in octanoylated-E2 subunits of PDH complexes to produce lipoylated proteins (Spalding and Prigge, 2010). It works together with the protein lipoyl(octanoyl) transferase [EC:2.3.1.181] (lipB) which transfers an octanoyl group from an octanoyl-acyl-carrier protein (octanoyl-ACP) produced through the type II fatty acid biosynthesis pathway, to the target apoprotein. Alternatively, octanoyl-E2 can be generated by the ATP-dependent ligation of free exogenous octanoate by the lipoate-protein ligase [EC:6.3.1.20] (Lpl). As lipA does not produce lipoate as a free acid (Spalding and Prigge, 2010), higher levels of lipA suggest greater levels of lipoylated-E2 subunits and in turn, suggest the involvement of lipoylated complexes such as PDH in the response against UV.

Following this line of evidence, there was also upregulation of proteins that seems to be linked to TPP biosynthesis through the supplying of substrates for hydroxymethylpyrimidine pyrophosphate (HMP-PP) and hydroxyethyl-thiazole phosphate (HET-P) synthesis (Rodionov et al., 2002). Because Act20 strain lacks an ortholog of a phosphomethylpyrimidine synthase [EC:4.1.99.17] (ThiC) to synthesize HMP-PP from intermediates of the purine biosynthesis pathway, exogenous HMP-P should be introduced into the cell through specific transporters complexes encoded by YkoCDE genes orthologs and then has to be phosphorylated by an hydroxymethylpyrimidine/phosphomethylpyrimidine kinase [EC:2.7.1.49 2.7.4.7] (ThiD) to yield HMP-PP (Figure 4). Results show that a homolog of the HMP/thiamine-import ATP-binding protein (YkoD) was significantly upregulated under the UV treatment, and also was the HMP/thiamine permease protein (YkoE) under FR after UV exposure (Figure 3A). Likewise, FR also showed high levels of YkoD but with lower FC_*difference*_ (Supplementary file 1). The high levels reached by the YkoD and YkoE in UV-exposed cultures suggest that the YkoCDE complex could be involved in UV response, probably by supplying substrates for HMP-PP and for TPP biosynthesis (Rodionov et al., 2002).

On the other hand, Act20 strain encodes genes to synthesize HET-P via glycine, cysteine, and glycolysis metabolism derivatives, such as iminoglycine, a thiocarboxy-sulfur-carrier protein (ThiS-COSH), and 1-deoxy-D-xylulose-5-phosphate (DXP) (Rodionov et al., 2002). In this process, glycine oxidase [EC:1.4.3.19] (ThiO) enzymatically oxidize glycine to produce iminoglycine (Figure 4 and Supplementary Figure 3). In turn, cysteine is use as SH groups donor to produce ThiS-COSH in a reaction catalyzed by cysteine desulfurase [EC:2.8.1.7] (iscS). Simultaneously, DXP is produced by coupling pyruvate and glyceraldehyde-3-phosphate by the enzyme 1-deoxy-D-xylulose-5-phosphate synthase (dxs). Then, iminoglycine, ThiS-COSH, and DXP are condensed by thiazole synthase [EC:2.8.1.10] (ThiG) to yield HET-P (Rodionov et al., 2002) (the condensation of these three compounds is represented as “thiazole biosynthesis pathway” in Figure 4) (see Supplementary Figure S3 for a complete TPP biosynthesis pathway).

In this context, the significant UV-induced upregulation of the substrate-binding subunit of the D-methionine transport system (metQ) and iscS protein (Figures 3A,B), as well as the ATP-binding subunit of L-cystine transport system [EC:7.4.2.1] (tcyC) (Supplementary file 1), suggest that the availability of cysteine as a SH group donor may be enhanced in UV-exposed cultures by direct uptake via TycABC transporters, or indirectly via MetNPQ transporters which import methionine that can be transformed into cysteine through the reverse *trans*-sulfuration pathway (Rodionov et al., 2004). In this pathway, methionine is first converted into *S*-adenosylmethionine (SAM) by *S*-adenosylmethionine synthetase [EC:2.5.1.6] (metK), and then into homocysteine via the SAM recycling pathway (Rodionov et al., 2004; Figure 4). Subsequently, homocysteine is converted into L-cystathionine by cystathionine beta-synthase [EC:4.2.1.22] (CBS) and then into cysteine by the action of a cystathionine gamma-lyase (CGL) (Figure 4; Rodionov et al., 2004). Act20 strain codes CBS and CGL genes in its genome clustered as an operon (Supplementary file 1).

Furthermore, the significant upregulation of Fructokinase (EC 2.7.1.4) (FrK) in UV-exposed cultures (Figure 3A), suggests that during UV-exposure intracellular fructose could be phosphorylated and converted into fructose-6-phosphate (Fruc-6P), a substrate of both glycolysis metabolism as well as the non-oxidative phase of the pentose phosphate pathway (Figure 4 and Supplementary Figure 1; Kelker et al., 1970; Binet et al., 1998; Caescu et al., 2004). Next, 6-phosphofructokinase (EC 2.7.1.11) (PFK) could catalyze the phosphorylation of Fruc-6P to fructose-1,6-bisphosphate (Fru-1,6PP), the first committed step of glycolysis, which then could be converted into glyceraldehyde-3-phosphate (G3P) by the action of fructose-bisphosphate aldolase, class II [EC:4.1.2.13] (FBA). As glycolysis proceed, DXP could be produced by coupling pyruvate and G3P through the action of dxs, which get into the thiazole biosynthesis pathway together with iminoglycine and ThiS-COSH yielding HET-P in a reaction catalyzed by ThiG. In addition, pyruvate yielded through glycolysis is also a substrate of the PDH complex (Spalding and Prigge, 2010).

Lastly, thiamin monophosphate (TP) is formed by coupling HMP-PP and HET-P catalyzed by thiamine-phosphate pyrophosphorylase [EC:2.5.1.3] (ThiE). At the next step, TP is phosphorylated by thiamine-monophosphate kinase [EC:2.7.4.16] (ThiL) to form thiamine pyrophosphate, the cofactor of E1-subunits of the PDH complexes (Rodionov et al., 2002). Eventually, TP and TPP can be dephosphorylated by thiamine phosphate phosphatase [EC: 3.1.3.100] (rsgA) to yield thiamine that, in turn, can be reused by the thiaminase [EC:3.5.99.2] (TenA) to produce HMP which can then be reintroduced into the TPP biosynthesis cycle.

In addition, the second functional module includes UV upregulated proteins taken from FR-UV and DR-UV comparisons (Figure 3C) which are intimately linked to the *de novo* synthesis and regeneration pathways of NAD+ (Rodionov et al., 2008), a cofactor whose function is associated to several reactions, including the functioning of the PDH complex (Spalding and Prigge, 2010). These proteins were PncA, PncB, nadA, nadD, and nudC (see annotations in Supplementary file 1).

As mentioned, the PDH complex can act as an antioxidant by associating DlaT and LpdC proteins with two alkylhydroperoxide reductase peroxyredoxins, AhpC and AhpD. In this system, the oxidized (lipoamide) and reduced (dihydrolipoamide) forms of lipoic acid bounded to DlaT, comprise a redox couple that can effectively extinguish harmful free radicals such as hydroxyl radicals, peroxyl radicals, or superoxide radicals. Briefly, AhpC reduces free radicals and is regenerated by oxidation of AhpD. AhpD is then reduced by oxidation of the dihydrolipoamide linked to DlaT, which in turn is regenerated by LpdC in a NADH-dependent reaction. Thus, AhpC, AhpD, DlaT and LpdC function as redox partners, and also, AhpD acts as a bridge between AhpC and the PDH complex suggesting that an increase in its abundance may increase the antioxidant activity of the complex (Figure 4; for a more detailed mechanism see Bryk et al., 2002; Spalding and Prigge, 2010).

In addition, UV exposure induced a significant increase in the abundance of MrpD protein, which is fundamental in the assembly and functioning of a membrane complex involved in maintaining intracellular homeostasis through extrusion of sodium ions and intrusion of protons (Ito et al., 2017). Thus, this protein may be included in the above hypothesized subsystem of damage tolerance. Also, the abundance levels of gvpK protein [involved in gas vesicle biosynthesis (Pfeifer, 2012)], a GntR transcriptional regulator [probably involved in uptake of citrate and related compounds as suggested from the genomic context analysis (Winnen et al., 2003)], and two uncharacterized proteins with DUF4229 and DUF3046 domains, respectively, were significantly increased by UV effect (Figures 3A, 4). Further work will be needed to characterize the exact functions of these proteins in the context of the UV-resistome.

### Effects of the Photorecovery Treatment on Molecular Processes After Ultraviolet-B Irradiation

**FIGURE 5:**
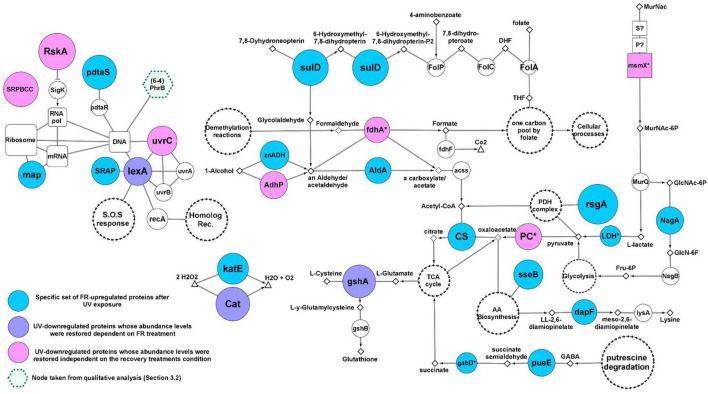
Molecular events involved in FR- response (see Figure 3C). Colored nodes indicate proteins whose abundance were significantly increased after UV exposure independent or dependently on FR (see legends in the picture). The size of colored nodes indicates abundance levels. Color-less nodes represent functionally related proteins encoded in the genome of strain Act20, but showing no significant FR-related changes in abundance. The size of color-less nodes are independent of their abundance levels. Rectangular nodes represent membrane transporters. White nodes with separate black arrow edges represent metabolic pathways. Diamonds represent substrates. Functional interactions among nodes are represented by borders: metabolic pathways/proteins–substrates interactions are represented by solid lines; metabolic pathways/proteins–products interactions are represented by arrows. This schema does not consider reversible reactions. All proteins are annotated in Supplementary file 1, sheet 5.

Upon UV-challenge, 26 proteins significantly decreased their abundance levels. In order to identify the cellular processes occurring once the UV stimulus has stopped and those stimulated by the photorecovery treatment, the proteins that decreased in abundance under UV irradiation and then restored their normal levels regardless of the recovery condition as well as those proteins that restored their levels by the effect of FR were studied (Figures 3C, 5; see annotations in Supplementary file 1).

Ultraviolet exposure significantly reduced the abundance of the RskA protein, which acts as a repressor of the extracytoplasmic transcriptional factor SigK (ECF19 family) through physical interactions (Staroń et al., 2009; Huang et al., 2015). Then, RskA restored its abundance during FR and DR treatments, suggesting that SigK may coordinate the expression of genes involved in the response against UV-B radiation. UvrC endonuclease involved in nucleotide excision repair of bulky DNA damages such as photoproduct was significantly downregulated under UV, but was restored to its original levels during FR and DR treatments suggesting that the UvrABC base excision repair complex may be involved in the repair of DNA damage caused by UV radiation. Besides, adhP (EC:1.1.1.1), fdhA (EC: 1.2.1.46), Pyruvate carboxylase (PC), and msmX are involved in anaplerotic reactions that supply substrates for the tricarboxylic acid (TCA) cycle through the catabolism of carbohydrates such as alcohols, aldehydes and pyruvate (Gerstmeir et al., 2003; Tanaka et al., 2003; Sauer and Eikmanns, 2005; Arndt and Eikmanns, 2007; Arndt et al., 2008; Marçal et al., 2009) and showed noticeable changes upon UV treatment. Their abundances were significantly decreased under UV exposure and increased later during the recovery treatments, suggesting that energy metabolism and amino acid precursor production from the TCA cycle is truncated by UV irradiation. The involvement of anaplerotic reactions in the recovery response was also supported by the upregulation of the zinc-dependent aldehyde dehydrogenase (znADH), the *Streptomyces aureofaciens* (AAD23400)-like aldehyde dehydrogenase (AldA), citrate synthase (CS), lactate dehydrogenase (LDH), gabD, and pueE proteins during FR treatment as well as AldH in DR (Sprušanský et al., 1999; Jaureguibeitia et al., 2007; Schneider and Reitzer, 2012; Figure 5).

Also, there was a restoration in abundance of both the catalase (Cat) belonging to the manganese cluster family and the glutamate-cysteine ligase gshA involved in thiol biosynthesis. In addition, an upregulation of a second catalase KatE belonging to the heme cluster family was found during FR (Figures 3C, 5), suggesting a high antioxidant activity under this experimental condition, probably in response to an increase in the concentration of free radicals as a consequence of UV irradiation as well as the re-establishment of the metabolic rate.

Another protein that was found significantly increased in FR was the SOS response repressor lexA. In contrast, the RecA protein remains strongly overregulated in DR indicating that white light may be enhancing the repair of damaged DNA in agreement to the photoproduct repair assay (Figure 1). Furthermore, the lower abundance values of RecA, radA, and priA proteins in FR treated cultures relative to DR and the upregulation of DNA gyrases and DNA ligases compared to Dt (Supplementary Figure 4) are indicative of a temporarily more active state of DNA repair induced by FR. These results support the existence of an efficient photo-induced mechanism of DNA repair possibly involving photolyases as suggested by the qualitative analysis and the photoproduct quantification assay. This finding agrees well with the abundance profile and the biological implications of lexA and RecA proteins (Khan and Kuzminov, 2012; Kreuzer, 2013).

Finally, due to their significantly high abundance values, three unrelated proteins were added to the FR response model, SulD, map, and pdtaS (Figure 5). SulD is an essential enzyme involved in the synthesis of pterins such as folate, dihydrofolate and tetrahydrofolate which are essential for cell growth and repair (Garçon et al., 2006).

The methionine amino peptidase (map) participates in the transduction mechanism and protein biosynthesis by removing N-terminal methionine from nascent proteins. Finally, PdtaS is a recently discovered sensor in Actinobacteria whose function and biological implications are currently under discussion. It is proposed that it is involved in the control of amino acid biosynthesis, ribosomal protein levels, and amino acid transfer RNA biosynthesis in a c-di-GMP dependent mechanism (Morth et al., 2005; Preu et al., 2012; Hariharan et al., 2019).

## Discussion

Our results confirm the high UV tolerance profile of *Nesterenkonia* sp. Act20 reported in previous works (Portero et al., 2019) and indicate photorecovery as critical strategy to ensure the viability of *Nesterenkonia* sp. cells exposed to high UV-B doses. Various physiological adaptations at the molecular level allow the organism to persist and survive in extreme environments (Thattai and Van Oudenaarden, 2004; Zenoff et al., 2006b; Albarracín et al., 2014; Portero et al., 2019).

The comparative proteomic assay of the strain Act20 revealed that proteins upregulated by UV-B exposure are part of three proposed UV-resistome subsystems: (i) damage tolerance and oxidative stress response; (ii) energy management and metabolic resetting; and (iii) DNA damage repair. Indeed, homologous recombination was shown to be the primary mechanism of DNA repair as suggested by the high levels of abundance of RecA and other recombinational proteins in UV exposed cultures (Kreuzer, 2013). While homolog recombination acts on single strand gaps and double strand breaks on DNA (secondary lesions), a point mutation (primary lesions) induced by UV irradiation can promote chromosomal fragmentation and single strand gaps when a replication fork advances across a primary lesion which then requires an efficient repair mechanism such as the RecA-dependent processes (Smith and Wang, 1989; Kowalczykowski, 2000; Morimatsu and Kowalczykowski, 2003; Khan and Kuzminov, 2012). Considering the protective mechanism against oxidative stress caused by UV exposure, the pyruvate dehydrogenase complex is the major system involved, as there was a significant increase in the abundance of proteins intimately related to its antioxidant activity and the production of the cofactors required for its function, mainly the lipA and AhpD proteins (Spalding and Prigge, 2010). Thus, despite the fact that at first glance ykoD, MetQ, tcyC, IscS, FrK, PncA, PncB, nadA, nadD, and nudC proteins seemed not to be related to the PDH complex, exhaustive literature mining and the and the review of curated databases indicate that these proteins might be linked to the supply of substrates for the production of cofactors such as thiamine pyrophosphate and NAD+ required by this complex (Figure 4). Equally, methionine imported by the MetQPN transporter is also necessary for the production of *S*-adenosylmethionine required by the lipoate synthase protein (LipA) which requires two equivalents of SAM to synthesize one equivalent of lipoic acid (Cicchillo et al., 2004), further linking also this set of proteins to the PDH complex. Likewise, it is also noteworthy to highlight the importance of the upregulation of Frk (EC:2.7.1.4) (Kelker et al., 1970; Binet et al., 1998; Caescu et al., 2004), as this protein accomplishes a dual function in the UV response model: it can supply substrates for thiamine pyrophosphate biosynthesis through the production of fructose-6P for glycolysis, and it is involved in the production of purine and pyrimidine metabolism intermediates through the non-oxidative phase of the pentose phosphate pathway (Supplementary Figure 1). In addition, intracellular fructose is the inducer of Frk expression (Kelker et al., 1970; Binet et al., 1998; Caescu et al., 2004). Since exogenous fructose is phosphorylated to fructose-1-phosphate when it enters the cell through the PTS FruAB II transport system, and that byproduct is neither a substrate or an inducer of Frk (ec 2.7.1.4) (Kelker et al., 1970; Binet et al., 1998; Caescu et al., 2004), the upregulation of Frk (EC:2.7.1.4) suggests that there is a consumption of intracellular fructose sources such as xylose or D-sorbitol (Figure 4). It should also be kept in mind that Frk remains abundant during recovery treatments. Thus, we consider that FrK (EC:2.7.1.4) is a key enzyme in the UV response, at least under oligotrophic conditions such as physiological solution and under the system tested here.

Upon exposure to UV radiation, the cell viability of Act20 cultures is recovered more efficiently under photorecovery treatment than in dark conditions. In accordance, the FR proteome overexpressed the SOS response repressor lexA, while RecA proteins remain highly abundant in DR-exposed cultures indicating that FR-exposed cultures were in a temporarily more advanced state of DNA repair than DR-cultures, since lexA and RecA proteins are intimately linked through a negative feedback circuit induced by the amount of damaged DNA (Kreuzer, 2013). This suggests the existence of an efficient photo-induced mechanism of DNA repair such as photolyases, as indicated by both the first analysis (in which there appears to be present a second type of photolyase under FR treatment, Figure 2B) and the photoproduct quantification assay in which there are significant reductions of photoproduct concentration in FR treatments (Figure 1). Replication events at DNA sites bearing cyclobutane pyrimidine dimers and pyrimidine (6-4) pyrimidone photoproduct can generate double strand breaks and single strand gaps in DNA (Smith and Wang, 1989; Kowalczykowski, 2000; Morimatsu and Kowalczykowski, 2003; Khan and Kuzminov, 2012). This induces the upregulation of RecA and other recombinational proteins, in turn triggering effective cleavage of lexA and thereby reducing its abundance. We reasoned that the stimulation of photolyase activity by the photorecovery treatment allows DNA lesions to be repaired before replication forks pass throughout these lesions, thus, preventing ssDNA gaps formation, chromosomal fragmentation, and RecA overexpression. In turn, decreased RecA abundance levels lead to the accumulation of lexA as occurs under FR conditions (Kowalczykowski et al., 1994; Nickoloff and Hoekstra, 2001; Kreuzer, 2013). Thus, photolyases seem to be critical elements of the UV-resistome of Act20 as they are the only light-driven enzymes detected in its genome capable of repairing damaged nucleotides in DNA. In addition, the substantial restoration of the RskA protein abundance levels after UV exposure and during recovery treatments suggests that the extra-cytoplasmic transcriptional factor sigK may coordinate the expression of stress response genes during UV irradiation (Staroń et al., 2009; Huang et al., 2015).

Finally, it is worth noting the remarkable similarity of Act20 molecular UV response mechanisms with those described for common nosocomial strains of the mycobacterial order in response to stress conditions such as the participation of pyruvate dehydrogenase complex through lipA and AhpD proteins (Tian et al., 2005; Shi and Ehrt, 2006; Spalding and Prigge, 2010), the increased levels of CdnL (Stallings et al., 2009; Garner et al., 2014), and the involvement of the RskA-sigK molecular system (Staroń et al., 2009; Huang et al., 2015). Act20 showed also a remarkable similarity to proteins involved in the anaplerotic reaction of the TCA cycle machinery for the catabolism of aldehydes and alcohols compared to the industrial strain *Corynebacterium glutamicum* (Sprušanský et al., 1999; Jaureguibeitia et al., 2007; Schneider and Reitzer, 2012). Besides, the critical involvement of lipA protein in the UV irradiation response of Act20 suggests greater levels of lipoic acid production, probably due to an increased abundance of free radicals. This supports the idea that lipoate supplementation strategies can contribute to combat oxidative stress (Chen et al., 2014). These notes are of paramount importance in the context of the current interest in finding strains capable of carrying out biotechnological processes under extreme conditions.

## Conclusion and Future Prospects

High-Altitude Andean Lakes are natural photobiology laboratories for exploring and monitoring *in situ* interactions between solar irradiation and the dynamics of biodiversity. They also provide us with model strains to perform *in vitro* assays and test light and specially UV effects on microbial physiology. In this work, we have compared the UV-resistance profile of two *Nesterenkonia* strains, one isolated from the HAAL (Act20) and the other in another extreme setting such as a desert soil from China (*N. halotolerans*). Our results indicated the UV-resistance superior phenotype of Act20, a clear reflection of the environmental conditions prevailing in the Puna region. This phenotype was based mainly in the Act20 ability to cope with much more photoproduct accumulation in its DNA but also with more efficient repairing systems triggered by light (photolyases). Current work is heading toward the heterologous expression and structural/functional characterization of both, putative 6,4 and CPD photolyases detected in Act20 genome and experimental proteome.

Moreover, the herein presented research represents an advance in the knowledge on the integral molecular response to UV-B radiation in an environmental bacterium. Through a comparative analytical approach and photoproduct measurements, Act20 UV-resistome was dissected. Unlike previous reports that studied particular molecular mechanisms (e.g., photorepair or oxidative damage response) involved in the UV-C response of lab strains such as *E. coli* we have studied Act20 response to UV-B in an integral manner configuring a functional network of tightly related molecular events detonated upon the radiation challenge. Moreover, there were consistent differences with the molecular events occurring in both repair treatments -with and without light. Indeed, this proteomic versatility may be a reflection on their original changing environment and of utmost importance for survival in this ecosystem, the highest UV irradiated environment on Earth. To our knowledge, this is the first work to show the UV-induced gene expression in a *Nesterenkonia* strain.

Finally, this work opens an avenue for biotechnological applications. As previously exposed, UV resistant microbes present a myriad of strategies to overcome the harmful radiation. In these strategies, molecules of diverse chemistry are produced and constitute excellent microbial metabolic reserves (i.e., extremolytes) that have been widely explored for industrial significance; however, their therapeutic implications remain to be investigated. That is the case for HAAL indigenous extremophiles and specially for *Nesterenkonia* sp. Act20 that produce biomolecules (i.e., ectoine, photolyase, carotenoids) adapted to their unusual living conditions that may represent valuable sources of novel bioproducts. This topic is intriguing and needs further investigations for reaching concrete applications.

## Data Availability Statement

The datasets presented in this study can be found in online repositories. The names of the repository/repositories and accession number(s) can be found in the article/Supplementary Material.

## Author Contributions

LP, FZ, and VA performed the experimental assays on DNA and protein manipulation. FZ performed the data analysis, interpretation and wrote the manuscript. VA designed and coordinated the research work and wrote the manuscript. TD performed the photoproduct measurements and subsequent analysis and wrote the manuscript. WG provided lab space, analyzed and interpreted data and wrote the manuscript. MF, WG, and VA obtained funding for the original project idea. MF performed sampling expeditions and provided strains for the present project. All authors contributed to the article and approved the submitted version.

## Conflict of Interest

The authors declare that the research was conducted in the absence of any commercial or financial relationships that could be construed as a potential conflict of interest.

## Publisher’s Note

All claims expressed in this article are solely those of the authors and do not necessarily represent those of their affiliated organizations, or those of the publisher, the editors and the reviewers. Any product that may be evaluated in this article, or claim that may be made by its manufacturer, is not guaranteed or endorsed by the publisher.

## Footnotes

1
https://massive.ucsd.edu/
