# Supplementary material for: Proteomic Signatures of Microbial Adaptation to the Highest Ultraviolet-Irradiation on Earth: Lessons From a Soil Actinobacterium
Source: Front Microbiol. 2022 Mar 15;13:791714. doi: 10.3389/fmicb.2022.791714 (PMC8965627; doi:10.3389/fmicb.2022.791714)
Supplement: Supplementary file 3 [file Data_Sheet_1.docx]

| **CH** | **AA** | **C & V** | **E** | **Others AA** | **Lip** | **Nucl** | **T & P** | **Aro** |
| --- | --- | --- | --- | --- | --- | --- | --- | --- |
| 91 | 93 | 93 | 94 | 91 | 90 | 94 | 84 | 94 |

**Table S1 –** Percentage of shared proteins (%) among Dt, UV, DR and FR protein datasets for each category of biomolecules, which in turn indicate the percentage of proteins that are replaced by the effect of the treatments.

**
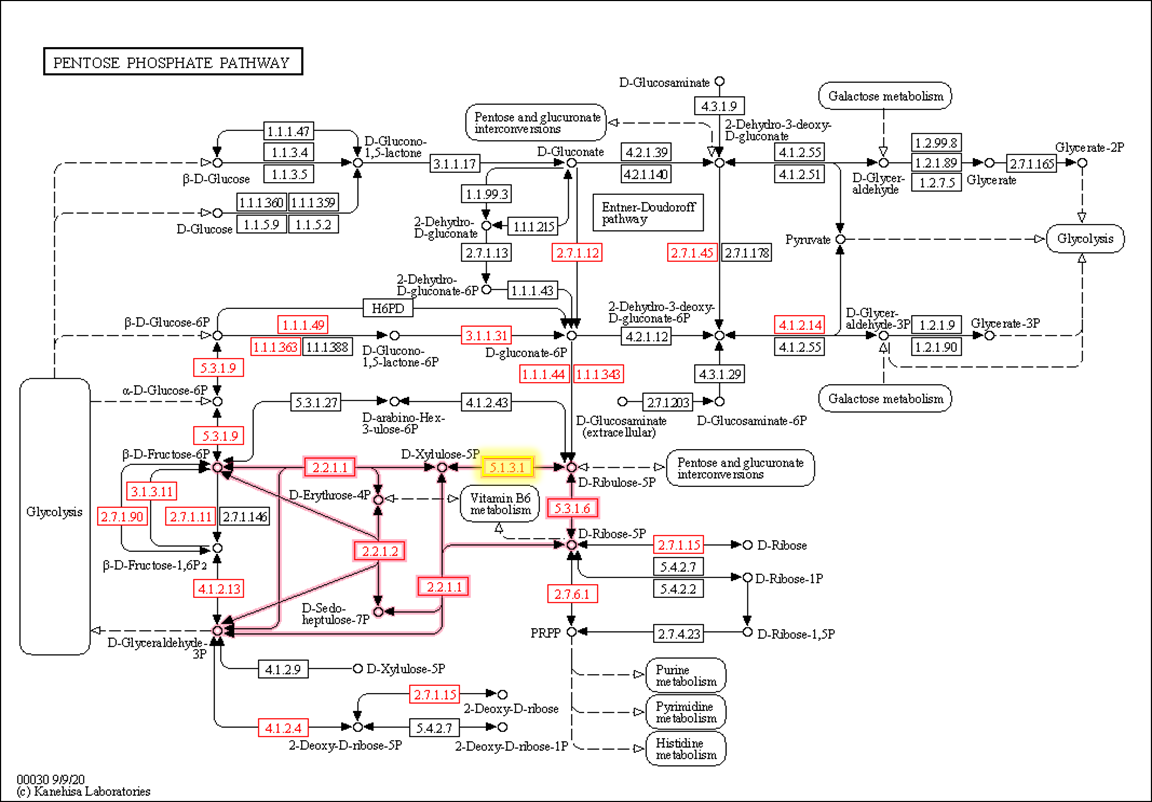
**

**Figure S1-** General view of pentose phosphate pathways from KEGG server (map00030). Red-marked items represent proteins encoded in *Nesterenkonia* sp. Act20 genome, whereas the yellow-marked item indicate the Ribulose-phosphate 3-epimerase (EC 5.1.3.1) (rpe) protein which was absent in Dt samples. Pink arrows indicate the non-oxidative phase of the pentose phosphate pathway.

**
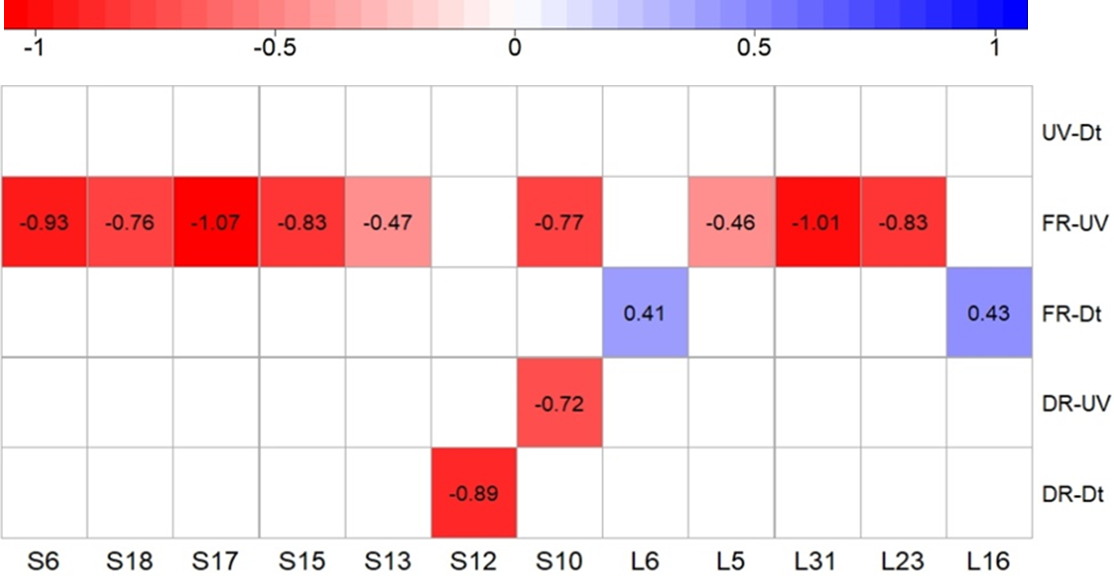
**

**Figura S2-** Heatmap comparing the abundance of ribosomal proteins with statistically significate values between treatments. Colors and their intensities represent the FC_difference_ for a particular ribosomal protein indicated at the bottom of the image. If the log mean abundance of a protein is higher in the first term of the comparison, then FC_difference_ get blue tones. If is higher in the second term, it gets red tones.


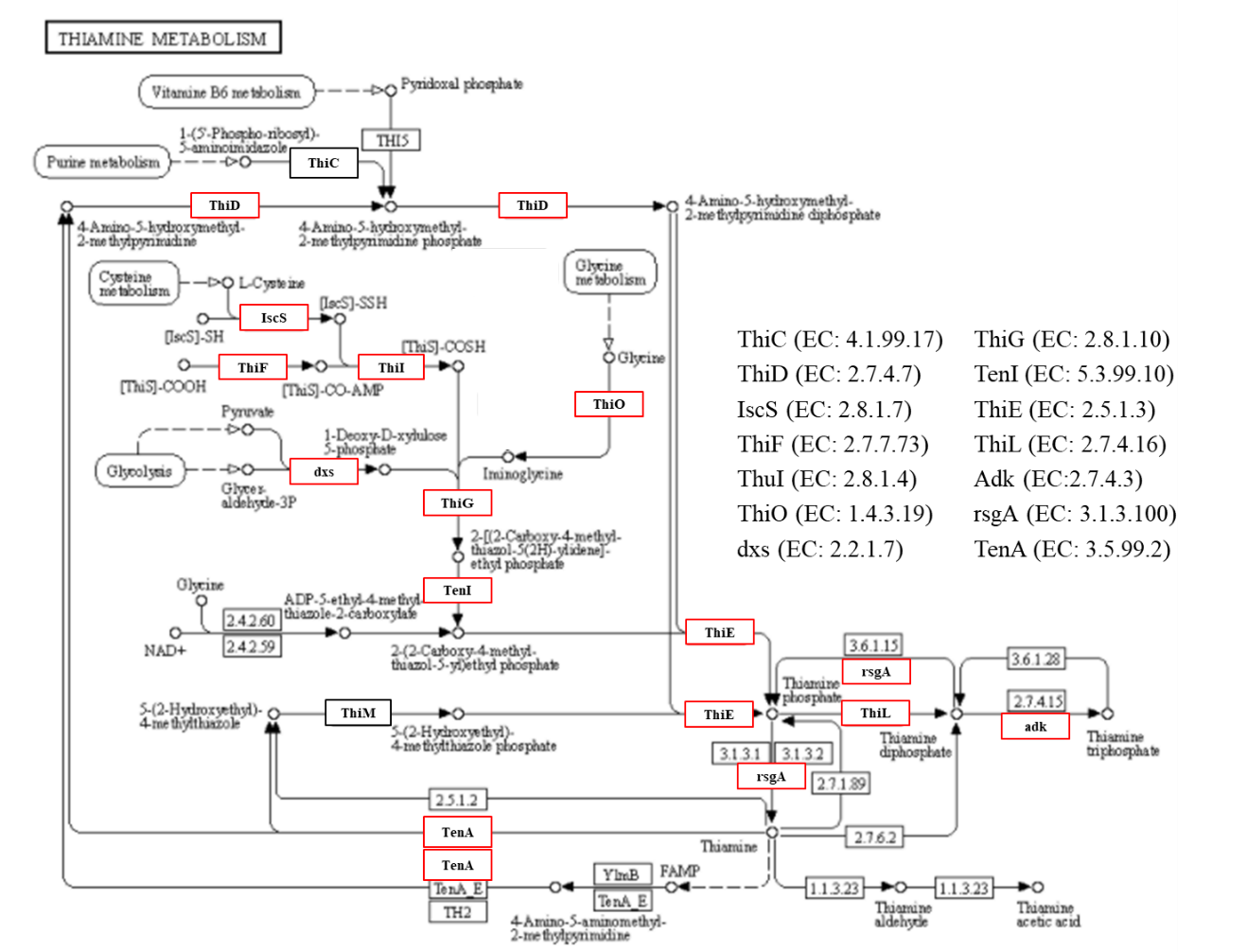


**Figura S3-** General view of thiamine biosynthesis pathways from KEGG server (map00730). Red-marked items represent proteins encoded in the Nesterenkonia sp. Act20 genome. The EC numbers of this proteins were replaced in the image by their canonical names in order to facilitate interpretation, but provided at the right side of the figure. Black-marked Items border represent proteins that apparently are not coded in the genome of Act20 strain. For pathway description see Rodionov et al., 2002, and map00730 in KEGG pathways.


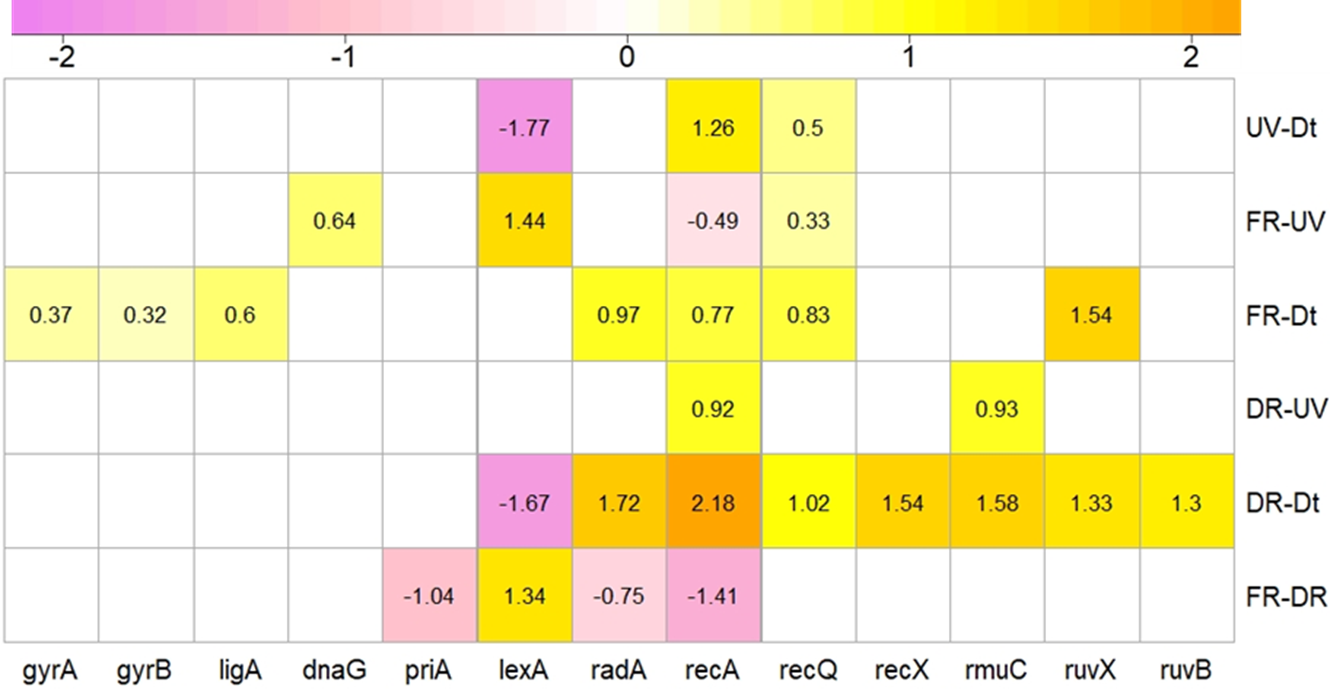


**Figure S4-** Heatmap comparing the abundance of proteins involved in homologous recombination and DNA repair between treatments. Colors and their intensities represent the FC_difference_ for a particular repair protein indicated at the bottom of the image. If the log mean abundance of a protein is higher in the first term of the comparison, then FC_difference_ get orange tones. If is higher in the second term, it gets pink tones.

**Note 1-** There are two definitions of "fold-change" (FC) in the literature (Witten & Tibshirani, 2007). The standard definition, referred to as FC_ratio_ (a), is denoted as the ratio of the average values of the normalized areas of a protein for two treatments. Following this definition, a protein will be over-regulated if the average abundance value for a protein in a given treatment is greater than twice the average abundance value for the same protein in another treatment. On the other hand, FC_difference_ (b), is denoted as the difference of the mean abundance values on a logarithmic scale in base 2 of a protein for two treatments. According to this definition, a protein in a given treatment will be over-regulated relative to another treatment if the FC_difference_ value exceeds the values 1 and -1.

**a)** FC*_ratio_* i = _i_ / _i_  **b)** FC*_difference_* i = _i_ - _i_


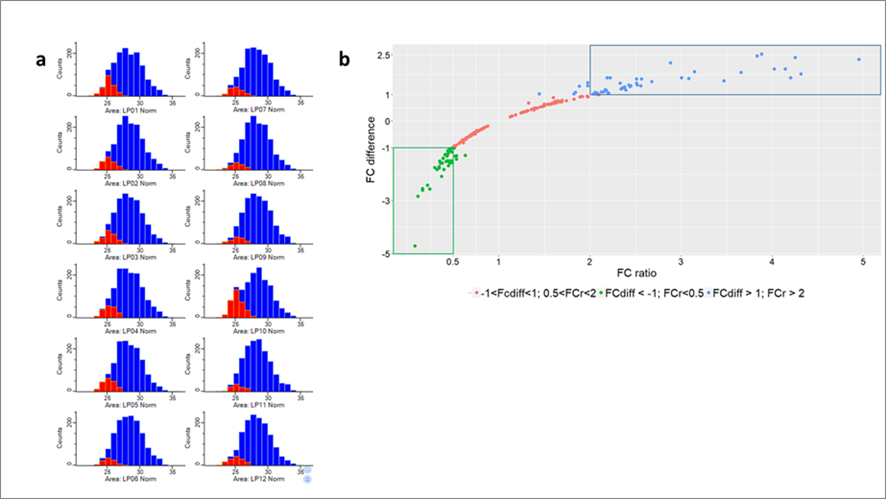


**Imputed values in proteomic dataset-** **a)** Invalid values (in red) imputed by the minimum values detected from the normal distribution of the whole dataset of each treatment for each biological replicate. **b)** Relationship between FC_ratio_ and FC_difference_ in the proteomic dataset (Supplementary file 1). It is observed that values of FC_ratio_ greater or less than twice (0.5>FC_ratio_<2) correspond to values of FC_difference_ greater than and less than 1 and -1, respectively.

**REFERECES**

Rodionov, D. A., Vitreschak, A. G., Mironov, A. A., & Gelfand, M. S. (2002). Comparative genomics of thiamin biosynthesis in procaryotes. New genes and regulatory mechanisms. Journal of Biological Chemistry, 277(50), 48949–48959. https://doi.org/10.1074/jbc.M208965200

Witten, D., & Tibshirani, R. (2007). A comparison of fold-change and the t-statistic for microarray data analysis. Analysis, 1776, 58–85. http://www-stat.stanford.edu/~tibs/ftp/FCTComparison.pdf
